# Study on the SEIQR model and applying the epidemiological rates of COVID-19 epidemic spread in Saudi Arabia

**DOI:** 10.1016/j.idm.2021.04.005

**Published:** 2021-04-18

**Authors:** Hamdy Youssef, Najat Alghamdi, Magdy A. Ezzat, Alaa A. El-Bary, Ahmed M. Shawky

**Affiliations:** a Mechanical Engineering Department, College of Engineering and Islamic Architecture, Umm Al-Qura University, Makkah, Saudi Arabia; b Department of Mathematics, Faculty of Applied Science, Umm Al-Qura University, Makkah, Saudi Arabia; c College of Science and Arts, Qassim University, Al Bukairiyah, Al Qassim, Saudi Arabia; d Arab Academy for Science, Technology and Maritime Transport, P.O. Box 1029, Alexandria, Egypt; e Science and Technology Unit (STU), Umm Al-Qura University, Makkah, Saudi Arabia; f National Committee for Mathematics, Academy of Scientific Research and Technology, Egypt

**Keywords:** COVID-19, Jacobian matrix, Lyapunov stability, Reproduction number, SEIR model, SEIQR model

## Abstract

This article attempts to establish a mathematical epidemic model for the outbreak of the new COVID-19 coronavirus. A new consideration for evaluating and controlling the COVID-19 outbreak will be constructed based on the SEIQR Pandemic Model. In this paper, the real data of COVID-19 spread in Saudi Arabia has been used for the mathematical model and dynamic analyses. Including the new reproductive number and detailed stability analysis, the dynamics of the proposed SEIQR model have been applied. The local sensitivity of the reproduction number has been analyzed. The domain of solution and equilibrium based on the SEIQR model have been proved using a Jacobian linearization process. The state of equilibrium and its significance have been proved, and a study of the integrity of the disease-free equilibrium has been carried out. The Lyapunov stability theorem demonstrated the global stability of the current model equilibrium. The SEIQR model has been numerically validated and projected by contrasting the results from the SEIQR model with the actual COVID-19 spread data in Saudi Arabia. The result of this paper shows that the SEIQR model is a model that is effective in analyzing epidemic spread, such as COVID-19. At the end of the study, we have implemented the protocol which helped the Saudi population to stop the spread of COVID-19 rapidly.

## Introduction

With the continuation of COVID-19 outbreaks, the number of infections is gradually growing, and this is because several factors increase the severity of COVID-19 infection and build obstacles to the management of diseases. Since scientists and researchers around the world are trying to set up a vaccine or an epidemic cure for control of such pandemics in the future, an infectious disease can be well identified and understood using mathematical models from a medical engineering context. This idea originated in 1927. Afterward, several different mathematical models for various diseases and infections were created. We refer to some critical studies (Gao et al., 2020; Goyal et al., 2019; Kumar et al., 2019; Martcheva, 2015a; Shah et al., 2020; Van den Driessche & Watmough, 2002). To define the dynamics of the transmission and to estimate the national and global spread of this disease, Wu et al. have implemented the Susceptible Exposed Infectious Recovered Model (SEIR) based upon the data recorded from December 31, 2019, to January 28, 2020. They also found that COVID-19 had a simple reproductive number of approximately 2.68 (Wu et al., 2020). Read et al. (Read et al., 2020) have reported a value of 3.1 for the basic reproductive number based on data fitting of the SEIR model, using an assumption of Poisson-distributed daily time increments. Imai et al.(Imai et al., 2020) developed a deterministic compartmental model that involved the disease’s clinical development, human epidemiological status, and engagement levels. The authors find that the reproductive control number may be as large as 6.47. That interaction techniques such as simplified touch tracing and quarantine would efficiently minimize the number of reproductive controls and the risk of transmission (Tang et al., 2020a). To predict the severity of the disease outbreak, Imai et al. (Imai et al., 2020) carried out a computer simulation of possible infectious tracks in Wuhan with an emphasis on communications between individuals. Their findings suggest that control measures must block more than 60% of transmission to avoid the outbreak. (Imai et al., 2020).

Guo et al. also developed a deep learning algorithm to evaluate the infectivity of the new coronavirus and to predict its future hosts. Their findings showed that maybe two animal hosts of this virus are bats and minks (Guo et al., 2020). Most of these models have highlighted the critical role played by direct, human-to-human transmission in this epidemic. They have shown that the majority of those infected have no interaction with the market in Wuhan and that the number of infections has risen steadily, and that the disease has spread to every province in China and more than 20 people. Many infected persons have a relatively long incubation period so that they are unaware of their infection for 10–14 days. They can easily spread the disease to others by direct contact during this period. On the other hand, the models published to date have not taken into consideration the role of the environment in COVID-19 transmission. Various further modeling research for the COVID-19 outbreak has also been carried out (Benvenuto et al., 2020; Chen et al., 2020; Din et al., 2020; Kucharski et al., 2020; Mangoni & Pistilli, 2020a; Nadim et al., 2020; Peng et al., 2020a; Rabajante, 2020; Read et al., 2020; Wang et al., 2020; Wu et al., 2020; Yang & Wang, 2020a).

Mathematical epidemiology is the topic of research at the population level of trends of health and disease. An infectious disease is characterized by the presence of a pathogenic microbial agent as a clinically obvious disorder. For modeling purposes, we define four different forms of transmission: directly, when the triggering pathogen is a person-to-person transmission of the pathogen; vector if the causative agent is a vector transmitted to a human; environmentally, if a person gets infected through interaction with an environmental pathogen; and vertically, if the pathogen is transmitted from mother to child by birth. Airborne and personal diseases are commonly considered to be transmitted directly when transmission takes place via any interaction between one person and another (Martcheva, 2015b). Mathematical modeling of infectious diseases is relevant and critical in the emergence of HIV epidemics. Since then, several models have been developed, studied, and applied to investigate the spread of infectious diseases. Mathematical modeling today makes a significant contribution to mathematics and public health (Hethcote, 2000; Martcheva, 2015b). The classic SEIR model is commonly used and accepted in many countries to assess the outbreak of the COVID epidemic. Since the mathematical model can draw simple and straightforward conclusions on the COVID-19 epidemic, a cascade of the SEIR models has been developed to explain the mechanisms of its infection source transmission, storage, and hosts for humans. (Chen et al., 2020; Youssef et al., 2020b, c). Babaei et al.(Babaei et al., 2021) constructed a mathematical model to examine the effect of quarantine on the spread of coronavirus.

This paper aims to construct a new COVID-19 model that is more applicable to cases in any country through mathematical analysis of the given model using a background of similar models with different considerations and further outflows between populations. Another aim is to research and learn the perfect procedures, controls, and techniques that could reduce the outbreak.

## Materials and methods

### Formulation of a novel coronavirus disease (SEIQR model)

**Fig. 1:**
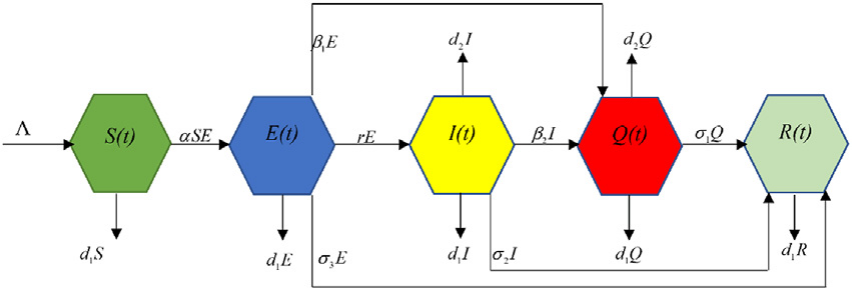
The flowchart of the proposed SEIQR model.

The population can be divided into five dynamic sub-populations or five groups that are represented in Fig. 1; during the propagation of COVID-19 in any region, and the following could be described:

The primary group S(t) is devoted to the susceptible population (individuals who are healthy but are able to contract the disease). For several diseases, the infected person may not become instantly contagious, but it is also called the latent phase. The pathogen needs time to spread and settle in the new host. The exposed time (latent) typically follows the sensitive point.

Group E(t) is also devoted to the exposed population or persons who are infected but not yet infectious. Group I(t) is intended for the confirmed infected population (individuals who contracted and are now ill, as well as infected persons).

The group Q(t) is dedicated to the quarantined population (hospitalized or isolated from the general population) (Maier & Brockmann, 2020).

The group R(t) is defined as the recovered population (individuals who have recovered and cannot contract the COVID-19 again), as in Fig. 1(Din et al., 2020; Maier & Brockmann, 2020; Mangoni & Pistilli, 2020b; Martcheva, 2015b; Pal et al., 2020; Peng et al., 2020b; Yang & Wang, 2020b; Youssef et al., 2020a; Youssef et al., 2020b, c).

The parameter α is defined as the transmission rate from a susceptible population to infected but not detected by the testing population. We consider the net inflow of the susceptible population at a non-negative rate Λ>0 per unit value of time (comprising new births and new residents). For any group, the outflow based on the natural death rate is defined by the non-negative rate $d_{1}$.

The total population size is N(t), which is defined as (Maier & Brockmann, 2020; Mangoni & Pistilli, 2020b; Martcheva, 2015b; Pal et al., 2020; Peng et al., 2020b; Yang & Wang, 2020b; Youssef et al., 2020a):

(1) N(t)=S(t)+E(t)+I(t)+Q(t)+R(t)

Starting with the group S(t), we have two outflows; a population flows out to the exposed group E(t) by the rate αS(t) (each one in S(t) can transfer the infection to αS(t), so the total number of outflow is equal to multiple αS(t)E(t), and the outflow of the natural death is$d_{1}S$. The group of exposed E(t) has only one inflow αE(t)S(t), while it has four outflows. The first outflow is the population that flows out to the group Q(t) by the rate of transmission $\beta_{1}$. The second outflow is the population that flows out to the recovery group directly without needing treatment by transmission rate of recovery $\sigma_{3}$. The third outflow is a population that flows out to the infected group I(t) with the transmission rate of infected r, and the fourth outflow is the population that experiences natural death by the transmission rate $d_{1}$ (Din et al., 2020; Maier & Brockmann, 2020; Mangoni & Pistilli, 2020b; Martcheva, 2015b; Pal et al., 2020; Peng et al., 2020b; Yang & Wang, 2020b; Youssef et al., 2020a).

For the group of confirmed infected population I(t), we have only one inflow, which comes from the group E(t), with the transmission rate r, while it has three outflows of population. The first outflow is the population that must go to the quarantine area Q(t) by the transmission rate $\beta_{2}$, and the second outflow comes from the population in which treatment has succeeded; individuals in this population can go out to the recovery group R(t) by recovery transmission rate$\sigma_{2}$. The last outflow from the infected group is the total death, which comes from natural death by transmission rate $d_{1}$ and death due to the COVID-19 virus by transmission rate of mortality $d_{2}$.

For the recovery population R(t), three inflows exist, and only one outflow. The first inflow comes from the quarantine area Q(t) by transmission rate of recovery $\sigma_{1}$, the second inflow is the population that comes out from the infected group by transmission recovery rate $\sigma_{2}$, and the third inflow is the population that flows out from the exposed area directly by transmission recovery rate $\sigma_{3}$. The only outflow from the recovering group is the death by natural transmission rate of mortality $d_{1}$. For the quarantine group Q(t), two inflows $\beta_{1}E\left(t\right)$ and $\beta_{2}I\left(t\right)$, and two outflows are present. The first outflow is the population flow out to the recovery group R(t) with transmission rate $\sigma_{1}$, while the second outflow is the total death, which comes from the natural death by transmission rate of death $d_{1}$, and by the transmission rate of death due to the COVID-19 virus $d_{2}$.

All inflows and outflows have been shown in the flowchart in Fig. 1, and the five groups can be converted into equations to formulate the following system of first-order ordinary non-linear differential equations (Din et al., 2020; Maier & Brockmann, 2020; Mangoni & Pistilli, 2020b; Martcheva, 2015b; Pal et al., 2020; Peng et al., 2020b; Yang & Wang, 2020b; Youssef et al., 2020a):

(2) $$\frac{dS\left(t\right)}{dt}=\Lambda -\alpha S\left(t\right)E\left(t\right)-d_{1}S\left(t\right)$$

(3) $$\frac{dE\left(t\right)}{dt}=\alpha S\left(t\right)E\left(t\right)-rE\left(t\right)-\beta_{1}E\left(t\right)-\sigma_{3}E\left(t\right)-d_{1}E\left(t\right)$$

(4) $$\frac{dI\left(t\right)}{dt}=rE\left(t\right)-\beta_{2}I\left(t\right)-\sigma_{2}I\left(t\right)-d_{1}I\left(t\right)-d_{2}I\left(t\right)$$

(5) $$\frac{dQ\left(t\right)}{dt}=\beta_{1}E\left(t\right)+\beta_{2}I\left(t\right)-\sigma_{1}Q\left(t\right)-d_{1}Q\left(t\right)-d_{2}Q\left(t\right)$$

(6) $$\frac{dR\left(t\right)}{dt}=\sigma_{1}Q\left(t\right)+\sigma_{2}I\left(t\right)+\sigma_{3}E\left(t\right)-d_{1}R\left(t\right)$$

We can simplify the above equations to be in the forms:

(7) $$\frac{dS\left(t\right)}{dt}=\Lambda -\alpha S\left(t\right)E\left(t\right)-d_{1}S\left(t\right)$$

(8) $$\frac{dE\left(t\right)}{dt}=\alpha S\left(t\right)E\left(t\right)-\varepsilon_{1}E\left(t\right)$$

(9) $$\frac{dI\left(t\right)}{dt}=rE\left(t\right)-\varepsilon_{2}I\left(t\right)$$

(10) $$\frac{dQ\left(t\right)}{dt}=\beta_{1}E\left(t\right)+\beta_{2}I\left(t\right)-\varepsilon_{3}Q\left(t\right)$$

(11) $$\frac{dR\left(t\right)}{dt}=\sigma_{3}E\left(t\right)+\sigma_{2}I\left(t\right)-d_{1}R\left(t\right)+\sigma_{1}Q\left(t\right)$$

where $\varepsilon_{1}=\left(r+\beta_{1}+\sigma_{3}+d_{1}\right)$, $\varepsilon_{2}=\left(\beta_{2}+\sigma_{2}+d_{1}+d_{2}\right)$ and $\varepsilon_{3}=\left(\sigma_{1}+d_{1}+d_{2}\right)$.

Theorem 1 ***(all solutions are definite positive)***

Each solution of the SEIQR model with its initial condition is a subset in the interval [0,∞) and {S(t),E(t),I(t),R(t),Q(t)}≥0 for all values 0≤t<∞.

Proof:

All the right-hand sides of the SEIQR model are completely continuous and locally Lipschitzian on ℝ. The solutions {S(t),E(t),I(t),R(t),Q(t)} with their initial conditions exist and are unique in the interval [0,∞) (Martcheva, 2015a).

It follows from equation (7) that:

(12) $$\frac{dS\left(t\right)}{dt}=\Lambda -\left(\gamma +d_{1}\right)\;S\left(t\right)$$

where. γ(t)=αE(t)

It can be re-written in the following form (Khan and Atangana, 2020):

(13) $$\frac{d}{dt}\left[S\left(t\right)\;\exp\left(d_{1}t+\int_{0}^{t}\gamma \left(\tau \right)d\tau \right)\right]=\Lambda \;\exp\left(d_{1}t+\int_{0}^{t}\gamma \left(\tau \right)d\tau \right)$$

Hence, we have

(14) $$S\left(t_{1}\right)\;\exp\left(d_{1}t_{1}+\int_{0}^{t_{1}}\gamma \left(\tau \right)d\tau \right)-S\left(0\right)=\int_{0}^{t_{1}}\Lambda \;\exp\left(d_{1}x+\int_{0}^{x}\gamma \left(\tau \right)d\tau \right)\;dx$$

For S(0)>0, it gives:

(15) $$S\left(t_{1}\right)\;\exp\left(d_{1}t_{1}+\int_{0}^{t_{1}}\gamma \left(\tau \right)d\tau \right)\geq \int_{0}^{t_{1}}\Lambda \;\exp\left(d_{1}x+\int_{0}^{x}\gamma \left(\tau \right)d\tau \right)\;dx$$

Thus, we obtain:

(16) $$S\left(t_{1}\right)\;\geq \exp\left(-d_{1}t_{1}-\int_{0}^{t_{1}}\gamma \left(\tau \right)d\tau \right)\times \int_{0}^{t_{1}}\Lambda \;\exp\left(d_{1}x+\int_{0}^{x}\gamma \left(\tau \right)d\tau \right)\;dx>0$$

Then, we get:

(17) $$S\left(t_{1}\right)\;>0$$

Similarly, it can be shown that E(t)>0,I(t)>0,Q(t)>0,andR(t)>0 (Khan and Atangana, 2020), which complete the proof.

Theorem 2 ***(the domain of solutions)***

All the solutions of the model structure that initiate in ${\mathbb{R}}_{+}^{5}$ are bounded inside the region ψ defined by. $\psi ={\left\{\left(S,E,I,Q,R\right)\in {\mathbb{R}}^{5}:0\leq N\left(t\right)\leq \frac{\Lambda }{d_{1}}\right\}}_{t\to \infty }$

Proof:

By differentiating both sides of equation (1), we get

(18) $$N^{\prime }\left(t\right)=S^{\prime }\left(t\right)+E^{\prime }\left(t\right)+I^{\prime }\left(t\right)+R^{\prime }\left(t\right)+Q^{\prime }\left(t\right)$$

Substituting from the model (7)–(11), we obtain

(19) $$N^{\prime }\left(t\right)=\Lambda -d_{1}N\left(t\right)-d_{2}\left(Q\left(t\right)+I\left(t\right)\right)$$

From Theorem 1, we have $d_{2}\left(Q\left(t\right)+I\left(t\right)\right)\geq 0$ ; hence, the following inequality is valid:

(20) $$N^{\prime }\left(t\right)+d_{1}N\left(t\right)\leq \Lambda$$

Then, we have

(21) $$N\left(t\right)\leq \left(N\left(0\right)-\frac{\Lambda }{d_{1}}\right)e^{-d_{1}t}+\frac{\Lambda }{d_{1}}$$

Then, when t→∞ we get the solution $N\left(t\right)\subset \left[0,\frac{\Lambda }{d_{1}}\right]$, which completes the proof.

### The equilibrium of the SEIQR model

To determine the equilibrium of this model, we set all the derivatives equal to zero and solve the system as follows (Martcheva, 2015a):

(22) $$S^{\prime }\left(t\right)=E^{\prime }\left(t\right)=I^{\prime }\left(t\right)=Q^{\prime }\left(t\right)=R^{\prime }\left(t\right)=0\to \left\{S,E,I,Q,R\right\}\equiv \text{constants}$$

which gives

(23) $$0=\Lambda -\alpha SE-d_{1}S$$

(24) $$0=\alpha SE-\varepsilon_{1}E$$

(25) $$0=rE-\varepsilon_{2}I$$

(26) $$0=\sigma_{1}Q+\sigma_{2}I+\sigma_{3}E-d_{1}R$$

(27) $$0=\beta_{1}E+\beta_{2}I-\varepsilon_{3}Q$$

From equation (25), we have

(28) $$E=\frac{\varepsilon_{2}}{r}I$$

From equation (24) when E≠0, we have

(29) $$S=\frac{\varepsilon_{1}}{\alpha }$$

Substituting from equations (28), (29) into equation (23), we get

(30) $$I=\frac{rd_{1}}{\varepsilon_{2}\alpha }\left(\frac{\alpha \Lambda }{d_{1}\varepsilon_{1}}-1\right)=\frac{rd_{1}}{\varepsilon_{2}\alpha }\left({ℜ}_{0}-1\right)$$

where

(31) $${ℜ}_{0}=\frac{\alpha \Lambda }{d_{1}\varepsilon_{1}}$$

Substituting from equation (30) into equation (28), we obtain

(32) $$E=\frac{d_{1}}{\alpha }\left({ℜ}_{0}-1\right)$$

Substituting from equations (30), (32) into equation (27), we get

(33) $$Q=\frac{\left(\beta_{1}\varepsilon_{2}+\beta_{2}r\right)d_{1}}{\alpha \varepsilon_{2}\varepsilon_{3}}\left({ℜ}_{0}-1\right)$$

Substituting from equations (30), (32), (33) in equation (26), we obtain

(34) $$R=\left(\frac{\left(\beta_{1}\varepsilon_{2}+\beta_{2}r\right)\sigma_{1}+\varepsilon_{3}\sigma_{2}r+\varepsilon_{2}\varepsilon_{3}\sigma_{3}}{\alpha \varepsilon_{2}\varepsilon_{3}}\right)\left({ℜ}_{0}-1\right)$$

Thus, at disease-free equilibrium (DFE) ${ℜ}_{0}=1$ gives E=I=Q=R=0 which leads to $\frac{\varepsilon_{1}}{\alpha }=\frac{\Lambda }{d_{1}}$, as in equations (23), (29), which agrees with the domain of solution in (21).

The number ${ℜ}_{0}$ is called the reproduction number (RBN) (Din et al., 2020; Martcheva, 2015b; Pal et al., 2020; Peng et al., 2020b; Yang & Wang, 2020b; Youssef et al., 2020a; Youssef et al., 2020b, c).

(35) $${ℜ}_{0}=\frac{\alpha \Lambda }{d_{1}\varepsilon_{1}}==\frac{\alpha \Lambda }{d_{1}\left(r+\beta_{1}+\sigma_{3}+d_{1}\right)}$$

Then, if ${ℜ}_{0}>1$ the system has a unique endemic equilibrium [7]:

(36) $$E_{0}^{*}=\left(S^{*},E^{*},I^{*},R^{*},Q^{*}\right)$$

where $S^{*}=\frac{\Lambda }{d_{1}}$, $E^{*}=\frac{d_{1}}{\alpha }\left({ℜ}_{0}-1\right)$, $\;I^{*}=\frac{d_{1}}{\alpha }\left({ℜ}_{0}-1\right)$, $Q^{*}=\frac{\left(\beta_{1}\varepsilon_{2}+\beta_{2}r\right)d_{1}}{\alpha \varepsilon_{2}\varepsilon_{3}}\left({ℜ}_{0}-1\right)$, and $R^{*}\;=\left(\frac{\left(\beta_{1}\varepsilon_{2}+\beta_{2}r\right)\sigma_{1}+\varepsilon_{3}\sigma_{2}r+\varepsilon_{2}\varepsilon_{3}\sigma_{3}}{\alpha \varepsilon_{2}\varepsilon_{3}}\right)\left({ℜ}_{0}-1\right)$.

Thus, the system has a unique disease-free equilibrium $E_{0}$ when ${ℜ}_{0}=1$ and has a unique endemic equilibrium $E_{0}^{*}$ when ${ℜ}_{0}>1$ (Martcheva, 2015b).

When ${ℜ}_{0}=0$, there is no transmission, where α=0.0. It can be interpreted as the number of secondary cases or the new infection rate (transmission rate at which the susceptible individual converted to an exposed individual) (Martcheva, 2015b).

The current proposed SEIQR model introduces a new and different reproduction number that is more sensitive to more parameters than other SEIQR models.

### Achieving equilibrium by applying a Jacobian matrix

To get the reproduction number ${ℜ}_{0}$ by using a Jacobian matrix method, we consider that the disease-free equilibrium (DFE) of the model SEIQR is acquired by setting E=I=R=Q=0 in equations (23), (24), (25), (26), (27). Hence, we obtain DFE in the form $E_{0}=\left(\frac{\Lambda }{d_{1}},0,0,0,0\right)$ (Din et al., 2020; Khan & Atangana, 2020; Martcheva, 2015a).

The Jacobian matrix of the SEIQR model takes the following form:

(37) $$J_{E_{0}}=\left[\begin{array}{lllll} -\alpha E-d_{1} & -\alpha S & 0 & 0 & 0\\ \alpha E & -\alpha S-\varepsilon_{1} & 0 & 0 & 0\\ 0 & r & -\varepsilon_{2} & 0 & 0\\ 0 & \beta_{1} & \beta_{2} & 0 & -\varepsilon_{3}\\ 0 & \sigma_{3} & \sigma_{2} & -d_{1} & \sigma_{1} \end{array}\right]$$

First, we will linearize the first two equations by using the Jacobian method. The first two equations have a disease-free equilibrium (DFE) situation when I=0→E=0 and $S=\frac{\Lambda }{d_{1}}$.

Hence, we consider that [5, 25,26]:

(38) $$F\left(S,E\right)=\Lambda -\alpha S\left(t\right)E\left(t\right)-d_{1}S\left(t\right)$$

(39) $$G\left(S,E\right)=\alpha S\left(t\right)E\left(t\right)-\varepsilon_{1}E\left(t\right)$$

Then, we have

(40) $$\left[\begin{array}{l} F\left(S,E\right)\\ G\left(S,E\right) \end{array}\right]=\left[\begin{array}{ll} \frac{\partial F}{\partial S} & \frac{\partial F}{\partial E}\\ \frac{\partial G}{\partial S} & \frac{\partial G}{\partial E} \end{array}\right]\left[\begin{array}{l} S\left(t\right)-S\left(0\right)\\ E\left(t\right)-E\left(0\right) \end{array}\right]=\left[\begin{array}{ll} -\alpha E\left(0\right)-d_{1} & -\alpha S\left(0\right)\\ \alpha E\left(0\right) & \alpha S\left(0\right)-\varepsilon_{1} \end{array}\right]\left[\begin{array}{l} S\left(t\right)-S\left(0\right)\\ E\left(t\right)-E\left(0\right) \end{array}\right]$$

By substituting from the equilibrium position, we obtain

(41) $$\left[\begin{array}{l} S^{\prime }\left(t\right)\\ E^{\prime }\left(t\right) \end{array}\right]=\left[\begin{array}{ll} -d_{1} & -\frac{\alpha \Lambda }{d_{1}}\\ 0 & \frac{\alpha \Lambda }{d_{1}}-\varepsilon_{1} \end{array}\right]\left[\begin{array}{l} S\left(t\right)-\frac{\Lambda }{d_{1}}\\ E\left(t\right) \end{array}\right]$$

Hence, the system of non-linear equations (7), (8) has been converted to the following linear system [5, 25,26]:

(42) $$\frac{dS\left(t\right)}{dt}=\Lambda -d_{1}S\left(t\right)-\frac{\alpha \Lambda }{d_{1}}E\left(t\right)$$

and

(43) $$\frac{dE\left(t\right)}{dt}=\left(\frac{\alpha \Lambda }{d_{1}}-\varepsilon_{1}\right)E\left(t\right)$$

For the complete system at equilibrium, the stability of the disease-free equilibrium (DFE) is given by the Jacobian matrix [5, 25,26]:

(44) $$J_{E_{0}}=\left[\begin{array}{lllll} -d_{1} & -\frac{\alpha \Lambda }{d_{1}} & 0 & 0 & 0\\ 0 & \frac{\alpha \Lambda }{d_{1}}-\varepsilon_{1} & 0 & 0 & 0\\ 0 & r & -\varepsilon_{2} & 0 & 0\\ 0 & \beta_{1} & \beta_{2} & 0 & -\varepsilon_{3}\\ 0 & \sigma_{3} & \sigma_{2} & -d_{1} & \sigma_{1} \end{array}\right]$$

By calculating the characteristic equation given by $\left|J_{E_{0}}-\lambda I_{5}\right|=0$, where λ is the eigenvalues parameter and $I_{5}$ is the identity matrix of order 5, then, the eigenvalues of the matrix $J_{E_{0}}$ take the following values:

(45) $$\left[\begin{array}{l} \lambda_{1}\\ \lambda_{2}\\ \lambda_{3}\\ \lambda_{4}\\ \lambda_{5} \end{array}\right]=\left[\begin{array}{l} -\varepsilon_{3}\\ -\varepsilon_{2}\\ -d_{1}\\ -d_{1}\\ \frac{\alpha \;\Lambda -d_{1}\varepsilon_{2}}{d_{1}} \end{array}\right]$$

### Condition of equilibrium (Hartman-Grobman theorem)

The Hartman-Grobman theorem says that the solutions of a square system of non-linear ordinary differential equations (7), (8), (9), (10) in a neighbourhood of a steady-state look “qualitatively” similar to the solutions of the linearized system near the point $E_{0}=\left(\frac{\Lambda }{d_{1}},0,0,0,0\right)$. This result holds only when the equilibrium is hyperbolic; that is when none of the eigenvalues of the matrix $J_{E_{0}}$ have zero real part (Martcheva, 2015b).

Thus, from equation (45) we obtain the following condition of equilibrium:

(46) $$\alpha \;\Lambda -d_{1}\varepsilon_{2}\neq 0$$

### The uniqueness of equilibrium condition

If the matrix $J_{E_{0}}$ is obtained from the linearization and is the Jacobian evaluated at equilibrium $DFE\left(E_{0}\right)=\left(\frac{\Lambda }{d_{1}},0,0,0,0\right)$, the condition $\left|J_{E_{0}}\right|\neq 0$ means that the equilibrium is isolated, which means there is a disk around it that does not contain other equilibria (Martcheva, 2015b).

Hence, from equation (44), we have

(47) $$\left|J_{E_{0}}\right|=\left|\begin{array}{lllll} -d_{1} & -\frac{\alpha \Lambda }{d_{1}} & 0 & 0 & 0\\ 0 & \frac{\alpha \Lambda }{d_{1}}-\varepsilon_{1} & 0 & 0 & 0\\ 0 & r & -\varepsilon_{2} & 0 & 0\\ 0 & \beta_{1} & \beta_{2} & 0 & -\varepsilon_{3}\\ 0 & \sigma_{3} & \sigma_{2} & -d_{1} & \sigma_{1} \end{array}\right|$$

which gives

(48) $$\left|J_{E_{0}}\right|=\varepsilon_{1}\varepsilon_{2}d_{1}\left(\alpha \Lambda -d_{1}\varepsilon_{1}\right)\neq 0$$

Thus, condition (46) is the only condition of the equilibrium of the SEIQR model.

Therefore, the unique equilibrium condition of the SEIQR model is:

(49) $$\frac{\alpha \;\Lambda }{d_{1}\varepsilon_{1}}-1\neq 0$$

The reproduction number (RBN) ${ℜ}_{0}=\frac{\alpha \Lambda }{d_{1}\varepsilon_{1}}$ is also unique (Martcheva, 2015b).

Theorem 3 ***(stability analysis of disease-free equilibrium)***

The SEIQR model $DFE\left(E_{0}\right)=\left(\frac{\Lambda }{d_{1}},0,0,0,0\right)$ is locally asymptotically stable under the condition ${ℜ}_{0}<1$ and unstable when ${ℜ}_{0}>1$ (Martcheva, 2015b).

Proof:

From the Jacobian matrix of system (44) which is defined at $DFE\left(E_{0}\right)=\left(\frac{\Lambda }{d_{1}},0,0,0,0\right)$ and the eigenvalues (45), we have

(50) $$\lambda_{1}=-\varepsilon_{4}<0,\;\lambda_{2}=-\varepsilon_{3}<0,\;\lambda_{3}=-\varepsilon_{1}<0,\;\lambda_{4}=-d_{1}<0$$

Thus, the system is locally stable when $\lambda_{5}=\frac{\alpha \;\Lambda -d_{1}\varepsilon_{1}}{d_{1}}<0$ which gives $\frac{\alpha \;\Lambda }{d_{1}\varepsilon_{1}}<1$, i.e., the stability condition takes the form:

(51) $${ℜ}_{0}=\frac{\alpha \;\Lambda }{d_{1}\varepsilon_{1}}<1$$

and the instability condition is:

(52) $${ℜ}_{0}=\frac{\alpha \;\Lambda }{d_{1}\varepsilon_{1}}>1$$

### Local sensitivity analysis of RBN $\left({ℜ}_{0}\right)$

Local sensitivity analysis is that examines the change in the output values that result from a change in one input value (parameter) (Youssef et al., 2020b, c).

The sensitivity or elasticity of quantity G concerning the parameter p is given by (Martcheva, 2015b):

(53) $${℘}_{G}^{H}=\frac{\partial G}{\partial H}/\frac{G}{H}=\pm \frac{\%\Delta G}{\%\Delta H}$$

The sensitivity of G concern H is positive if G is increasing concerning H and negative if G is decreasing concerning H.

Applying formula (53) into the reproduction number ${ℜ}_{0}$ which takes the form:

(54) $${ℜ}_{0}=\frac{\alpha \;\Lambda }{d_{1}\varepsilon_{1}}=\frac{\alpha \Lambda }{d_{1}\left(r+\beta_{1}+\sigma_{3}+d_{1}\right)}$$

Then,

(55) $${℘}_{{ℜ}_{0}}^{\alpha }=\frac{\partial {ℜ}_{0}}{\partial \alpha }/\left(\frac{{ℜ}_{0}}{\alpha }\right)=1>0$$

(56) $${℘}_{{ℜ}_{0}}^{\beta_{1}}=\frac{\partial {ℜ}_{0}}{\partial \beta_{1}}/\left(\frac{{ℜ}_{0}}{\beta_{1}}\right)=-\frac{\;\beta_{1}}{\varepsilon_{1}}<0$$

(57) $${℘}_{{ℜ}_{0}}^{\sigma_{3}}=\frac{\partial {ℜ}_{0}}{\partial \sigma_{3}}/\left(\frac{{ℜ}_{0}}{\sigma_{3}}\right)=-\frac{\;\sigma_{3}}{\varepsilon_{1}}<0$$

(58) $${℘}_{{ℜ}_{0}}^{r}=\frac{\partial {ℜ}_{0}}{\partial r}/\left(\frac{{ℜ}_{0}}{r}\right)=-\frac{\;r}{\varepsilon_{2}}<0$$

(59) $${℘}_{{ℜ}_{0}}^{d_{1}}=\frac{\partial {ℜ}_{0}}{\partial d_{1}}/\left(\frac{{ℜ}_{0}}{d_{1}}\right)=-\frac{\;d_{1}+\varepsilon_{1}}{\varepsilon_{1}}<0$$

The fact that ${℘}_{{ℜ}_{0}}^{\beta_{1}}=-\frac{\;\beta_{1}}{\varepsilon_{1}}$, ${℘}_{{ℜ}_{0}}^{\sigma_{3}}=-\frac{\;\sigma_{3}}{\varepsilon_{1}}$, ${℘}_{{ℜ}_{0}}^{r}=-\frac{\;r}{\varepsilon_{1}}$, and ${℘}_{{ℜ}_{0}}^{d_{1}}=-\frac{\;d_{1}+\varepsilon_{1}}{\varepsilon_{1}}$ means that a 1% increase in each one of $\left(\beta_{1},\sigma_{3},r,d_{1}\right)$ will produce $\left(\frac{\;\beta_{1}}{\varepsilon_{1}},\frac{\;\sigma_{3}}{\varepsilon_{1}},\frac{\;r}{\varepsilon_{1}},\frac{\;d_{1}+\varepsilon_{1}}{\varepsilon_{1}}\right)\%$ a decrease in ${ℜ}_{0}$, respectively. From relation (55), ${℘}_{{ℜ}_{0}}^{\alpha }=1$ it implies that a 1% increase α will produce a rise of 1% in ${ℜ}_{0}$ (Martcheva, 2015a).

### Global stability of equilibria of the SEIQR model (Lyapunov stability theorem)

One of the most used is the Lyapunov function. Lyapunov functions are scalar functions that may be used to prove the global stability of equilibrium. Lyapunov states that if a function V(x) is globally positively definite and radially unbounded, and its time derivative is globally negative, $V\left(x\right)<0\;for\;all\;x\neq x^{*}$ then the equilibrium $x^{*}$ is globally stable for the autonomous system $x^{\prime }=f\left(x\right)$, and V(x) is called a Lyapunov function (Martcheva, 2015b).

Theorem 5 ***(global stability)***

The SEIQR model $DFE\left(E_{0}\right)=\left(\frac{\Lambda }{d_{1}},0,0,0,0\right)$ is globally stable of the disease-free equilibrium under the condition ${ℜ}_{0}<1$.

Proof:

We will consider the SEIQR model on the space of the first three variables only (S,E,I). If the disease-free equilibrium for the first three equations is globally stable, then (Q,R)→0, and the disease-free equilibrium for the full SEIQR model is globally stable.

We construct the Lyapunov function on ${\mathbb{R}}_{+}^{3}$ in the following form (Martcheva, 2015b):

(60) $$V=\gamma \left(S-S^{*}-S^{*}\;\ln\left(\frac{S}{S^{*}}\right)\right)+\frac{E}{\varepsilon_{1}}+\frac{I}{r}$$

where γ is a parameter will be determined later, and $S^{*}=\frac{\Lambda }{d_{1}}$.

equation (60) shows that, at the disease-free equilibrium $\left(S^{*}=\frac{\Lambda }{d_{1}},0,0\right)$ , V=0.

Now, we have to show that V>0 for all $\left(S,E,I\right)\geq \left(\frac{\Lambda }{d_{1}},0,0\right)$.

equation (60) can be re-written as follows:

(61) $$V=\gamma S^{*}\left(\frac{S}{S^{*}}-1-\ln\left(\frac{S}{S^{*}}\right)\right)+\frac{E}{\varepsilon_{1}}+\frac{I}{r}$$

The first term is positive for any value of $S/S^{*}$, and the other two terms are also non-negative, so. V>0

Now, we take the derivative of equation (60), we obtain:

(62) $$V^{\prime }=\gamma \left(1-\frac{S^{*}}{S}\right)S^{\prime }+\frac{E^{\prime }}{\varepsilon_{1}}+\frac{I^{\prime }}{r}$$

Substituting from the first three equations of the SEIQR model and using equation (29), we obtain

(63) $$V^{\prime }=2\Lambda \gamma -\frac{\gamma \alpha \varepsilon_{3}}{r}SI-d_{1}\gamma S-\frac{\Lambda^{2}\gamma }{Sd_{1}}+\;\frac{\gamma \alpha \Lambda \varepsilon_{3}}{d_{1}r}I+\frac{\alpha \varepsilon_{3}}{\varepsilon_{1}r}SI-\frac{\varepsilon_{3}}{r}I$$

We choose $\gamma =\frac{1}{\varepsilon_{1}}$, then we have:

(64) $$V^{\prime }=-\frac{\Lambda }{\varepsilon_{1}}\left(\frac{d_{1}S}{\Lambda }+\frac{\Lambda }{Sd_{1}}-2\right)+\frac{\varepsilon_{3}}{r}I\left({ℜ}_{0}-1\right)$$

Since ${ℜ}_{0}<1$ then, the last term is non-positive.

For the first term, consider $\frac{d_{1}S}{\Lambda }=y$, then the term inside the brackets takes the form $\left(y+\frac{1}{y}-2\right)=\frac{{\left(y-1\right)}^{2}}{y}>0$, and now we have two possibilities. The first one is at the equilibrium point, where we have $S=S^{*}=\frac{\Lambda }{d_{1}}$ , and it gives y=1, then the first term completely vanishes, then we have the last term only, which is already non-negative. Thus, $V^{\prime }<0$.

The second possibility is y≠1, then the two terms are non-positive. Thus $V^{\prime }<0$.

Hence, we have $V^{\prime }<0$ for every $\left(S\left(t\right),E\left(t\right),I\left(t\right)\right)\;\geq \left(\frac{\Lambda }{d_{1}},0,0\right)$.

Therefore, by the Lyapunov theorem, the disease-free equilibrium is globally asymptotically stable for the system of the SEIQR model in all (Martcheva, 2015b).

### Solutions for the system of the SEIQR model

After linearization of the system of the SEIQR model, the system takes the form:

(65) $$\frac{dS\left(t\right)}{dt}=\Lambda -d_{1}S\left(t\right)-\frac{\alpha \Lambda }{d_{1}}E\left(t\right)$$

(66) $$\frac{dE\left(t\right)}{dt}=\left(\frac{\alpha \Lambda -d_{1}\varepsilon_{1}}{d_{1}}\right)E\left(t\right)$$

(67) $$\frac{dI\left(t\right)}{dt}=rE\left(t\right)-\varepsilon_{3}I\left(t\right)$$

(68) $$\frac{dR\left(t\right)}{dt}=\sigma_{3}E\left(t\right)+\sigma_{2}I\left(t\right)-d_{1}R\left(t\right)+\sigma_{1}Q\left(t\right)$$

(69) $$\frac{dQ\left(t\right)}{dt}=\beta_{1}E\left(t\right)+\beta_{2}I\left(t\right)-\varepsilon_{3}Q\left(t\right)$$

We assume the initial conditions of the above system take the form:

(70) $${\left\{S\left(t\right),E\left(t\right),I\left(t\right),Q\left(t\right),R\left(t\right)\right\}|}_{t=0}=\left\{S\left(0\right),E\left(0\right),I\left(0\right),Q\left(0\right),R\left(0\right)\right\}$$

To solve the above system, we start to solve equation (66) as follows:

(71) $$E\left(t\right)=E\left(0\right){\mathrm{e}}^{\gamma_{1}t}$$

where $\gamma_{1}=\frac{\alpha \Lambda -d_{1}\varepsilon_{1}}{d_{1}}$.

Then, by substituting from equation (71) into equation (67), we get

(72) $$\frac{dI\left(t\right)}{dt}+\varepsilon_{3}I\left(t\right)=rE\left(0\right){\mathrm{e}}^{\gamma_{1}t}$$

By solving the above equation, we get the infection function in the form:

(73) $$I\left(t\right)=\left(I\left(0\right)-\gamma_{2}\right)e^{-\varepsilon_{3}t}+\gamma_{2}e^{\gamma_{1}t}$$

where $\gamma_{2}=\frac{rE\left(0\right)}{\gamma_{1}+\varepsilon_{3}}$.

We can solve the first equation of the system by using equation (71)

(74) $$\frac{dS\left(t\right)}{dt}-d_{1}S\left(t\right)=\Lambda -\frac{\alpha \Lambda E\left(0\right)}{d_{1}}{\mathrm{e}}^{\gamma_{1}t}$$

After solving the above equation, we get

(75) $$S\left(t\right)=\left(S\left(0\right)+\gamma_{3}-\frac{\Lambda }{d_{1}}\right)e^{-d_{1}t}+\left(\frac{\Lambda }{d_{1}}-\gamma_{3}{\mathrm{e}}^{\gamma_{1}t}\right)$$

where $\gamma_{3}=\frac{\Lambda }{d_{1}}\left(\frac{\alpha E\left(0\right)}{d_{1}+\gamma_{1}}-1\right)$.

By inserting equations (71), (73), (75) into equations (68), (69), we obtain a system of two ordinary differential equations on R(t)andQ(t), which has been solved by using MAPLE software. We could not write the final forms of the two functions R(t)andQ(t) because it contains many long terms.

## Results

To verify the model SEIQR, we will apply it to the real data regarding the COVID-19 outbreak in Saudi Arabia. COVID-19 has been in Saudi Arabia since March 3, 2020. Cases continued to be discovered in small numbers until the beginning of April, and then the number of detected cases increased. Therefore, we decided in this study to consider April 1, 2020, as the real beginning of the spread of the COVID-19 epidemic in Saudi Arabia.

We used tables of statistics issued from the Saudi Ministry of Health (Health, 2020) and the daily official statement issued by the ministry as well as Wikipedia (Saudi_Arabia, 2020), which also depends on the ministry’s website and other websites that would announce these statistics.

Another source of these data is the “Saudi Centre for Disease Prevention and Control (Control, 2020)." We used the official website of the General Statistics Authority of Saudi Arabia for more information about the kingdom’s population, mortality rate, and population growth rate.

**Fig. 2:**
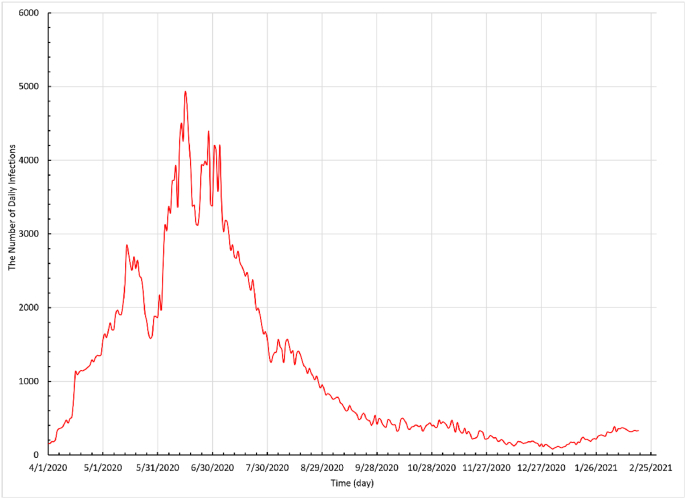
The real number of daily infections in Saudi Arabia between 4/1/2020 and 2/17/2021.

**Fig. 3:**
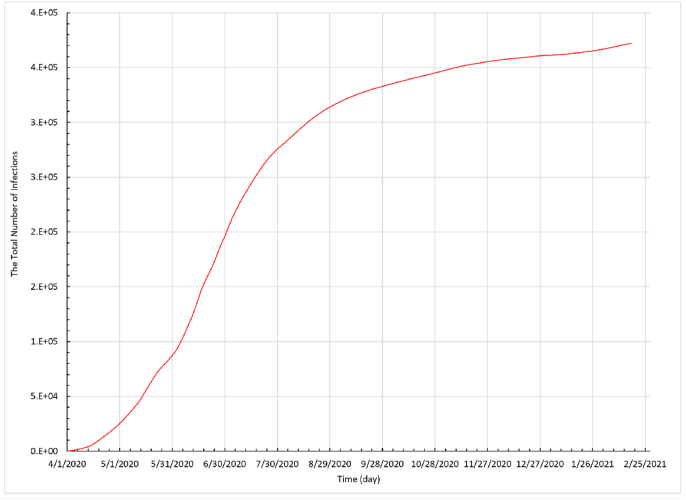
The total number of infections in Saudi Arabia between 4/1/2020 and 2/17/2021.

To study the spread of COVID-19 in Saudi Arabia, we will represent the curve of the number of daily infections and the time series curve of the total number of infections, as shown in Fig. 2, Fig. 3.

Fig. 2 shows that the number of daily cases on April 1, 2020, was 157 infections, and it reached 4919 infections on June 15, 2020, and between the two numbers, the curve passed through many up and down variations. After June 15, 2020, the number of daily infections has taken a decreasing curve with some changes up and down until February 17, 2021.

Fig. 3 shows that the total number of infections at the same interval started with 157 infections and reaches an accumulated amount of 139,506 infections on June 15, 2020, and an accumulated amount 372,200 number of infections on February 17, 2021. Therefore, we will use these data through the present model SEIQR to discern whether there is a convergence between the model results and the real data.

### Applying the SEIQR model to Saudi Arabia data of the spread of Covid-19

According to the official data of Saudi Arabia, we have the initial data, which is considered the initial conditions of the system based on the SEIQR model, as in Table 1(Control, 2020; Health, 2020; Saudi_Arabia, 2020):

**Table 1:** The initial conditions of the model SEIQR.

Time (day)	S(0)	E(0)	I(0)
April 1, 2020	34,218,169	$1.0\times 10^{4}$	157
June 16, 2020	34,297,479	$1.0\times 10^{5}$	4757

The total number of new births of Saudi children and new residents Λ≈2300 person/day and the rate of natural deaths is approximately 1030 people/day, which results in $d_{1}\approx 3\times 10^{-5}$. Some of the other parameters have been calculated, estimated, or assumed, as in Table 2.

**Table 2:** The values of parameters in SEIQR (Control, 2020; Din et al., 2020; Gerberry & Milner, 2009; Health, 2020; Jumpen et al., 2009; Khan and Atangana, 2020; Pal et al., 2020; Peng et al., 2020b; Saudi_Arabia, 2020; Tang et al., 2020b).

Parameter	Value 1/4/2020–15/6/2020	Value 16/6/2020–17/2/2021	Background
Λ	2300	2300	Calculated
$\beta_{1}$	0.15	0.2	Assumed
$\beta_{2}$	$1.0\times 10^{-5}$	$1.0\times 10^{-5}$	Assumed
$\sigma_{1}$	$1.0\times 10^{-5}$	0.02	Calculated
$\sigma_{2}$	$1.0\times 10^{-5}$	$1.0\times 10^{-5}$	Estimated
$\sigma_{3}$	$1.0\times 10^{-3}$	0.05	Estimated
r	0.00325	0.05	Calculated
$d_{1}$	$3.0\times 10^{-5}$	$3.0\times 10^{-5}$	Calculated
$d_{2}$	$3.423\times 10^{-7}$	$3.423\times 10^{-7}$	Calculated
α	$2.64\times 10^{-9}$	$1.18\times 10^{-9}$	Estimated
${ℜ}_{0}$	1.262>1	0.42<1	Calculated

By using MAPLE software, we get the results that indicate the number of daily infections as outcomes of the SEIQR model in the interval from April 1, 2020, to June 15, 2020. The value of the parameter α in that interval (the rate of transmission from susceptible population to infected in Saudi Arabia) was $\alpha =2.64\times 10^{-9}$, and the reproduction number RBN $\left({ℜ}_{0}\right)$ was ${ℜ}_{0}=1.262>1$. In other words, the transmission rate at which the susceptible individual converted to an exposed individual was higher than one, which means the spread of COVID-19 was unstable within the studied interval from April 1, 2020, to June 15, 2020.

After June 15, 2020, the value of the parameter α in that interval was $\alpha =1.18\times 10^{-9}$, and the reproduction number RBN $\left({ℜ}_{0}\right)$ was ${ℜ}_{0}=0.42<1$. In other words, the transmission rate at which the susceptible individual converted to an exposed individual was smaller than one, which means the spread of COVID-19 was stable within the studied interval.

**Fig. 4:**
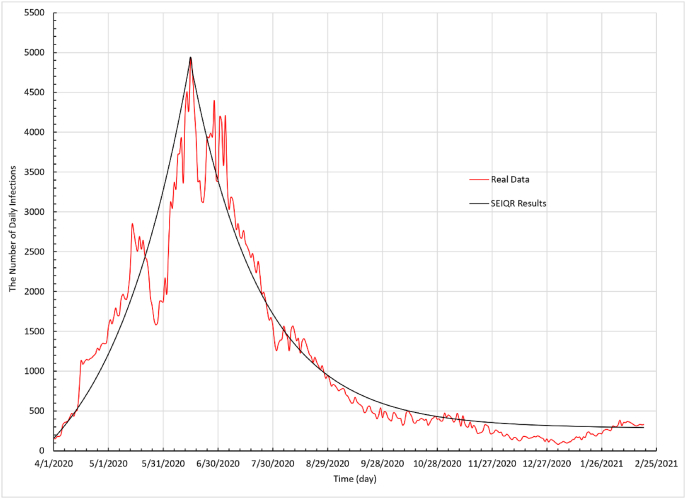
The number of daily infections based on the SEIQR model against the real data in Saudi Arabia between 4/1/2020 and 2/17/2021.

Fig. 4 shows the number of daily infections based on the SEIQR model against the real data in Saudi Arabia between 4/1/2020 and 6/15/2020 with the value of the rate of transmission from susceptible populations to infected in Saudi Arabia $\alpha =2.64\times 10^{-9}$ which gives the value of RBN ${ℜ}_{0}=1.262$ and between 6/16/2020 and 2/17/2021 with the value of the rate of transmission from susceptible populations to infected in Saudi Arabia $\alpha =1.18\times 10^{-9}$ which gives the value of RBN ${ℜ}_{0}=0.42$. It is noted that the curve which comes as results from the SEIQR model work as trends to the curve that belong to the real data. It makes the results due to applying the SEIQR model are close to the real data.

**Fig. 5:**
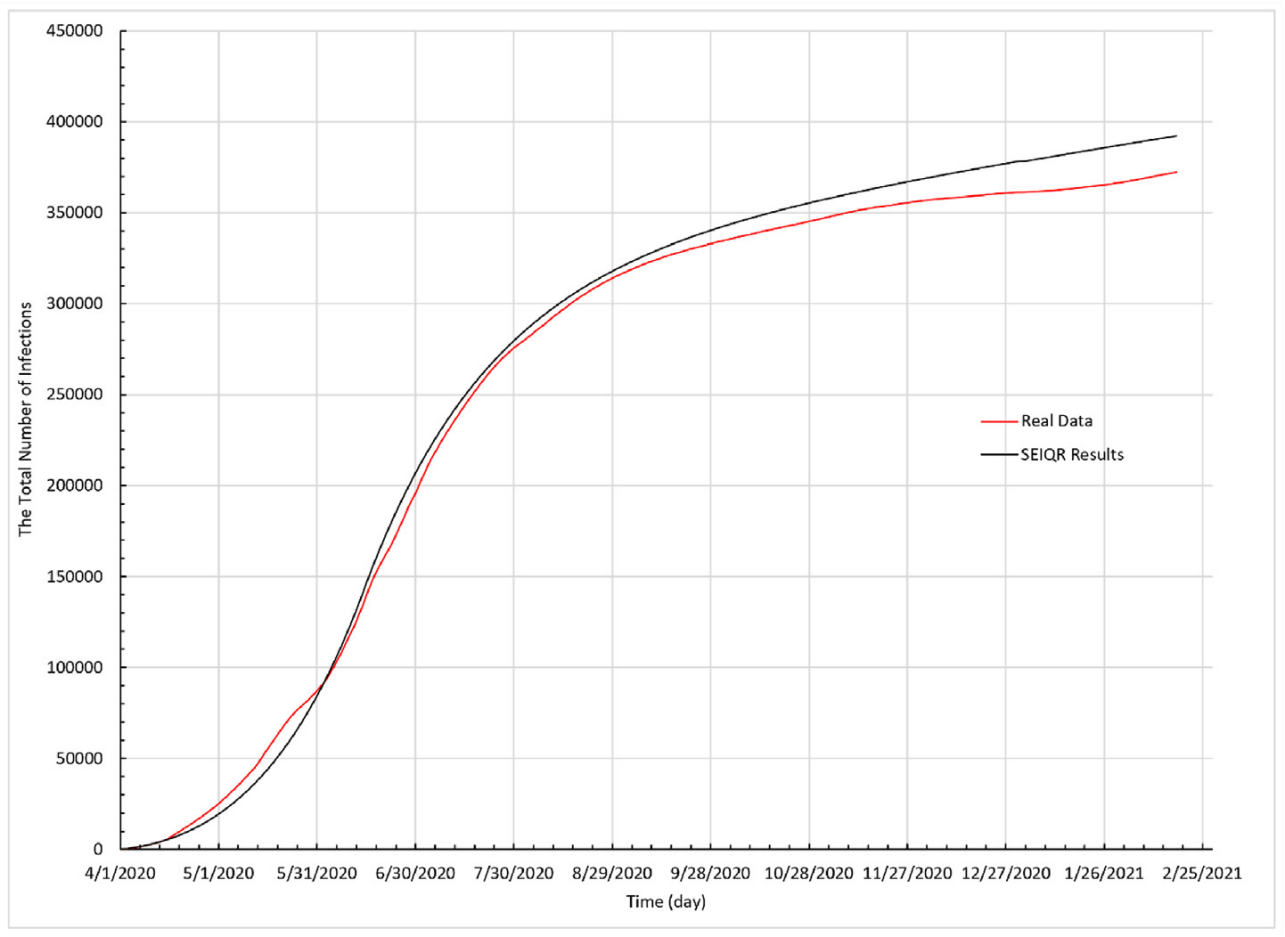
The total number of infections based on the SEIQR model against the real data in Saudi Arabia between 4/1/2020 and 2/17/2021.

To illustrate the convergence between the results of the proposed model SEIQR and the real results, we displayed Fig. 5 in which the cumulatively infected numbers within the same interval 4/1/2020 and 2/17/2021. This figure proves the success of the proposed SEIQR model.

## Discussions

According to the SEIQR model results and the real situation, we can conclude what happened and what makes us could halt the spread of COVID-19 in Saudi Arabia:

1. Decrease the value of the transmission rate from the susceptible population to infected but not detected by testing the population to be in the following interval $\alpha \leq 1.18\times 10^{-9}$.
2. Increase the value of the transmission coefficient from infected but not detected by testing population to a quarantine population $\beta_{1}$ to be $\beta_{1}\geq 0.2$, which means expanding the detection work and the need to isolate infected people in compulsory quarantine areas as an example.
3. Increase the value of the transmission rate from the quarantine population to the recovery population $\sigma_{1}$ to be $\sigma_{1}\geq 0.02$, which means that we must apply the successful treatment on the quarantine population and help them to recover.
4. Increase the value of the transmission rate from infected and not detected individuals by testing the population to recovery $\sigma_{3}$ to be $\sigma_{3}\geq 0.05$ by using a successful treatment.
5. Increase the value of infected but not detected individuals by checking the population to infected population for treatment r to be $r_{1}\geq 0.05$, which means we have to offer the more effective and accurate methods of diagnosis to find out the confirmed infections.

## Conclusion

In this work, we developed a new mathematical epidemic SEIQR model for the outbreak of the new COVID-19 coronavirus. This pandemic model offers a new method for evaluating and handling the COVID-19 epidemic. In Saudi Arabia, actual COVID-19 data have been used to validate the findings of this new model. The results show that the SEIQR model is a useful model for studying the spread of epidemics in Saudi and other countries, such as COVID-19.

The current model introduced a new and different reproduction number which is more sensitive to more parameters than the past SEIQR models.

Five steps were the perfect procedure, and thorough advice was implemented to help the population of Saudi Arabia slow the spread of COVID-19. Prevention is one of the key targets of this procedure rather than treatment.

The other main problem that helps to delay COVID-19 spread is to remain at home and to keep sick people in an isolated area or a protected location.

Finally, we need a safe and effective treatment of people with infections and vitamins, tonics, and supplements to protect non-infected people.

## Declaration of competing interest

The authors declare that they have no known competing financial interests or personal relationships that could have appeared to influence the work reported in this paper.
